# Polyethylene Glycol-Based Synthetic Hydrogel Sealant for Filtration Bleb Leaks: An In Vivo and Histologic Study

**DOI:** 10.1167/tvst.9.6.24

**Published:** 2020-05-22

**Authors:** Tatsuo Nagata, Yukinori Harada, Mikki Arai, Tatsuo Hirose, Hiroyuki Kondo

**Affiliations:** 1 Department of Ophthalmology, University of Occupational and Environmental Health, Fukuoka, Japan; 2 Arai Eye Clinic, Fukuoka, Japan; 3 The Schepens Eye Research Institute, Harvard Medical School, Boston, MA, USA

**Keywords:** management of bleb leak, post-glaucoma surgery, hydrogel sealant, PEG sealant, FocalSeal

## Abstract

**Purpose:**

To evaluate the efficacy of polyethylene glycol (PEG)-based synthetic sealant for closing bleb leaks after glaucoma filtration surgery.

**Methods:**

Tube shunt surgery that included implantation of a 22-gauge indwelling catheter and intraoperative mitomycin C was performed in the left eyes of 11 New Zealand white rabbits. Seven days postoperatively, all filtration blebs were perforated with an 18-gauge needle to create a bleb hole. In six rabbits, the holes were covered with the sealant and irradiated with blue-green light for 60 seconds; in the five control rabbits, the holes were untreated. For 3 weeks after the tube shunt surgery, the eyes were checked for bleb leaks, and the intraocular pressure (IOP) was measured in both eyes. Finally, the operated eyes were enucleated for histologic examination.

**Results:**

The bleb leaks stopped in the eyes in which sealant was used and persisted in the other eyes. The sealant preserved the bleb function; the IOPs in these eyes were significantly (*P* < 0.05) lower than the right eyes that did not undergo surgery. Hematoxylin and eosin staining showed that the holes were closed and covered with conjunctival epithelial cells in the eyes in which sealant was applied; the holes were open in the control eyes. Immunohistochemical staining showed that the bleb holes in which the sealant was applied had fewer inflammatory cells.

**Conclusions:**

The PEG sealant has the potential to seal bleb leaks effectively.

**Translational Relevance:**

Application of the PEG sealant can be used as adjunct therapy for bleb leaks in glaucoma surgery.

## Introduction

Leakage of filtration blebs after glaucoma surgery often leads to serious complications, including choroidal effusions, hypotony maculopathy, suprachoroidal hemorrhages, shallow anterior chambers, peripheral anterior synechiae, endophthalmitis, and bleb failure. Several techniques have been reported to manage bleb leaks,^1^ including pressure patching, bandage soft contact lenses, remodeling filtration blebs,^2^ aqueous suppressants, compression sutures, conjunctival sutures, argon laser,^3^^,^^4^ autologous blood injections,^5^^,^^6^ and tissue glue. Although fibrin glue^7^^–^^9^ or cyanoacrylate tissue glue^10^^–^^12^ are alternatives to the use of sutures to manage bleb leaks after trabeculectomy, they have not been used widely.

A sealant containing polyethylene glycol (PEG) was recently granted a Conformité Européenne mark that permits its use in Europe to seal suture lines as an adjunct to the standard closure techniques during arterial and venous reconstruction in vascular surgery, and during elective pulmonary resection for visceral pleural air leaks.^13^ PEG compounds can be engineered to form adherent hydrogel coatings with varying absorption times, consistencies, and flexibilities depending on the indications for use. Moreover, the PEG-based synthetic hydrogel sealant has been used during vitrectomy to close scleral wounds^14^ and patch retinal breaks in rhegmatogenous retinal detachments in a rabbit model effectively and safely.^15^^,^^16^

The current in vivo study was designed to evaluate the effectiveness of the PEG sealant for stopping postoperative bleb leaks after glaucoma surgery. The effect on the conjunctival hole and biocompatibility were also examined histologically.

## Methods

### Materials

The US Food and Drug Administration (FDA) approved the PEG sealant, FocalSeal (Genzyme Corporation, Cambridge, MA), to limit air leakage after pulmonary resection.^17^ Although the original formulation was clear and flexible (viscosity, 0.7–1.4 Pa·s), the sealant is polymerized by visible light from a xenon arc lamp (the wavelength: 450–500 nm, blue-green light) in 40 to 60 seconds. The FocalSeal sealant consists of an ethylene glycol-oligotrimethylene carbonate copolymer end-capped with acrylate esters. The solution also contains triethanolamine (90 mM) and eosin Y as a photoinitiator. The sealant serves as a firmly adherent hydrogel that can seal air or fluid leaks.^17^^,^^18^ The sealant is melted away by hydrolysis for 1 to 6 months, and releases biocompatible components that are metabolized or cleared by the kidneys.^18^ The residual PEG sealant in the syringe was frozen at –80°C and can be reused. Genzyme Corporation provided the sealant used in this study.

### Surgical Procedure

The study adhered to the ARVO Statement for the Use of Animals in Ophthalmic and Vison Research.

**Figure 1.**
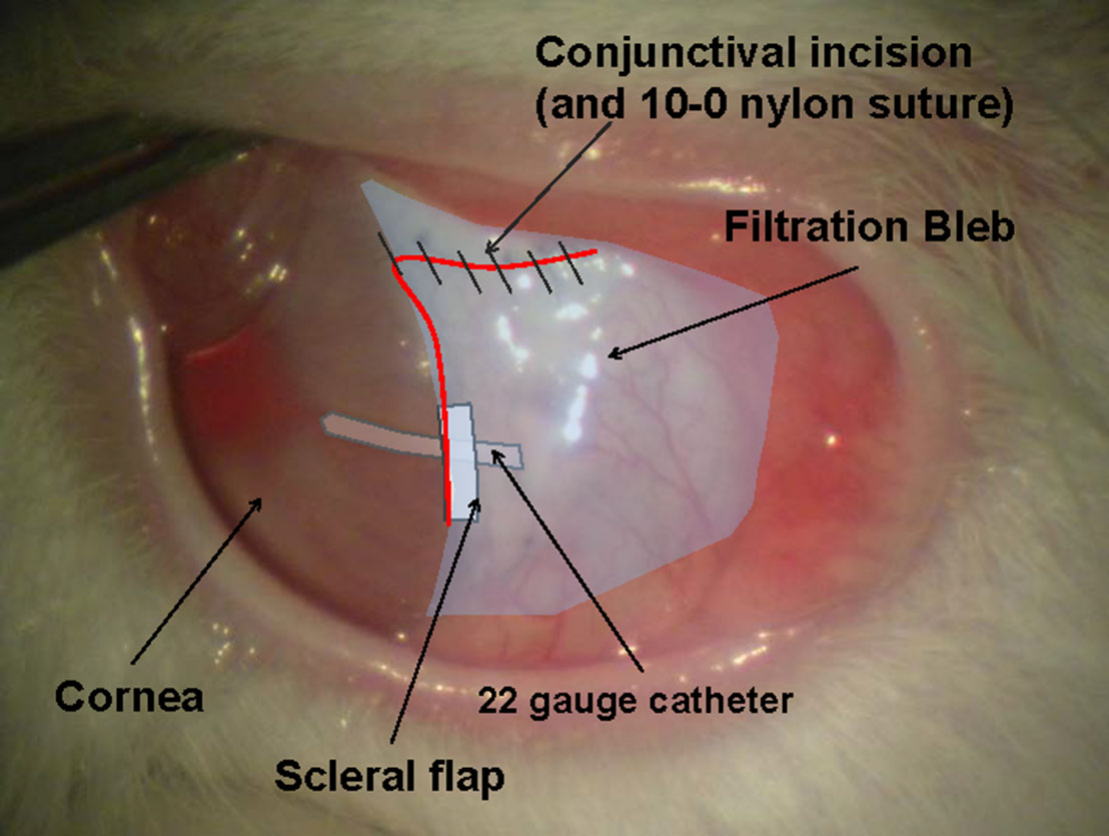
Photograph and sketch of the eye after the completion of the surgery. A fornix-based conjunctival flap is created in the left eye of the rabbit. Tube shunt surgery using a 22-gauge indwelling catheter with one suture and a partial-thickness scleral flap is performed in the sclera in the posterior upper limbus. The conjunctival incision is closed with six to eight end-to-end sutures.

A tube shunt surgery using a 22-gauge indwelling catheter (φ 0.7 × 5.0 mm) was performed in the left eyes of 11 New Zealand white rabbits (Biotek Co. Ltd., Saga, Japan) (weight, 1.6–2.4 kg). The rabbits were divided into two groups: five rabbits served as controls, and six rabbits received the PEG sealant. Preoperatively, the intraocular pressure (IOP) was measured using a Tono-Pen XL Applanation Tonometer (Reichert Technologies, Depew, NY), and the rabbits then were anesthetized with an intramuscular xylazine hydrochloride (8 mg/kg) and continuous intravenous infusion of thiopental (30 mg/kg/h). A traction suture was placed through the cornea. A fornix-based conjunctival flap was created. A cellulose sponge soaked with mitomycin C, 0.5 mg/mL, was placed between the sclera and the conjunctival flap for 5 minutes. The site was irrigated thoroughly with saline solution after the sponges were removed. A fornix-based filtration bleb was created with the tube shunt surgery (Fig. 1), and a partial-thickness scleral flap (3 × 2 mm) was created at the posterior upper limbus sclera (corneoscleral junction). A shunt catheter was sutured to the sclera with 10-0 nylon to prevent deviation. The conjunctival incision was closed with 10-0 nylon end-to-end sutures. The postoperative IOP was measured after topical anesthesia of oxybuprocaine, and an intramuscular injection of xylazine hydrochloride was induced.

**Figure 2.**
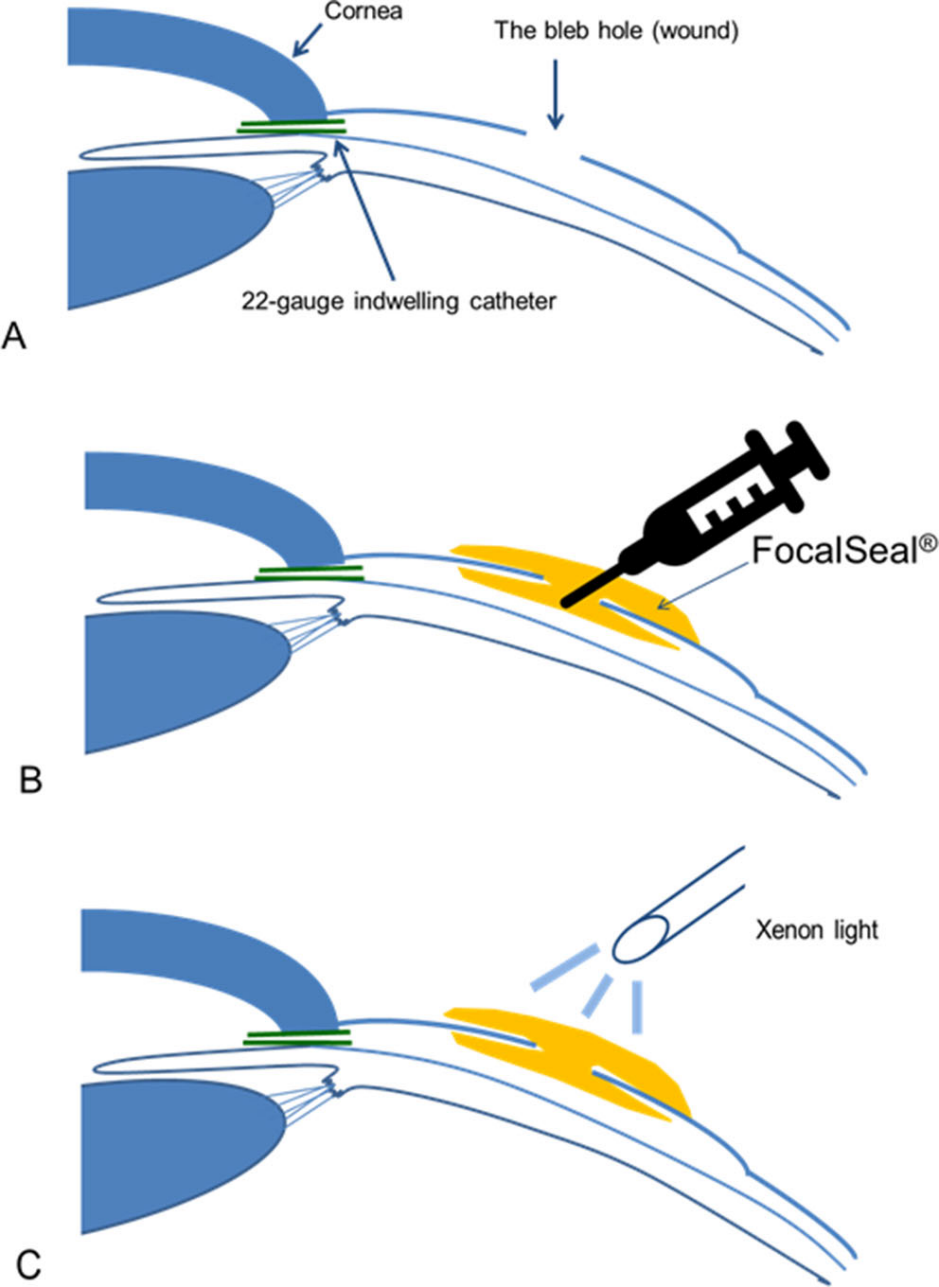
(A, B) Diagram of the process for sealing bleb holes with the PEG sealant. PEG sealant was applied to the subconjunctival space under the hole. Then the hole was covered with the sealant. (C) The PEG sealant is polymerized by 60-second application of xenon light.

Seven days after the tube shunt surgery, with the 11 rabbits under general anesthesia, a hole 1.2 mm in diameter was made in the highest part of the bleb using an 18-gauge needle (Fig. 2A). The holes were untreated in the five control rabbits. In the other six rabbits, the hole was closed immediately using the PEG sealant. The area around the hole was first dried as much as possible using sterile cotton-tipped applicators and surgical sponges (Medical Quick Absorber; Inami & Co., Ltd., Tokyo, Japan), then 0.03 to 0.05 mL of the PEG sealant was applied to the hole using a 5-cc syringe attached to a 27-gauge blunt needle (Fig. 2B). The sealant covering the hole was irradiated with a xenon arc light for 60 seconds (Fig. 2C). All blebs were checked for leaks, and the IOP was measured in all rabbits. We also checked the bleb leakage by forceful intraocular irrigating solution (BSS PLUS; Alcon, Ft. Worth, TX) injection into the anterior chamber. A moistened fluorescein strip was applied to determine the presence of leaks from the conjunctival holes. When it was difficult to distinguish the leaks with the fluorescein strip, 0.06% trypan blue was injected into the anterior chamber.

### Histology

The rabbits were euthanized with an overdose of thiopental sodium 21 days after the tube shunt surgery, and the eyes were enucleated, fixed in 2% paraformaldehyde and 2.5% glutaraldehyde solution, dehydrated in a series of increasing alcohol concentrations, and embedded in paraffin. Specimens from each bleb that included the hole were cut into 4-μm-thick sections; stained with hematoxylin and eosin (HE), Masson trichrome, anti-α-smooth muscle actin (α-SMA), proliferating cell nuclear antigen, and vimentin for immunochemistry; and examined under a light microscope (Axioplan; Carl Zeiss, Oberkochen, Germany).

### Statistical Analysis

Statistical analysis of IOPs was performed using SAS software (SAS Institute, Inc., Cary, NC). The Wilcoxon *t*-test was used to compare the IOPs between the control and the operated eyes. *P* < 0.05 was considered significant.

## Results

The PEG sealant stopped the bleb leaks and closed the holes. No leakage was observed at any time after the procedure in any eyes in which the PEG sealant was used. The PEG sealant prevented bleb leakage despite a challenge with injection of BSS PLUS into the anterior chamber.

The IOPs in the left eyes in which the sealant was applied were consistently significantly lower compared with each right eye in which no surgery was performed (*P* < 0.05, Wilcoxon *t*-test; see Table). Because of the bleb leakages, the IOPs in the control eyes were consistently lower than the IOPs in the sealant application eyes; IOPs in the control were too low to measure by Tono-Pen (Reichert Technologies) (<5 mm Hg). The leaks continued in the five control eyes for more than 2 weeks.

**Table.** The Mean IOP (mm Hg) in Each Eye Group Before and 1, 3, 7, 14, and 21 Days After the Tube Shunt Surgery

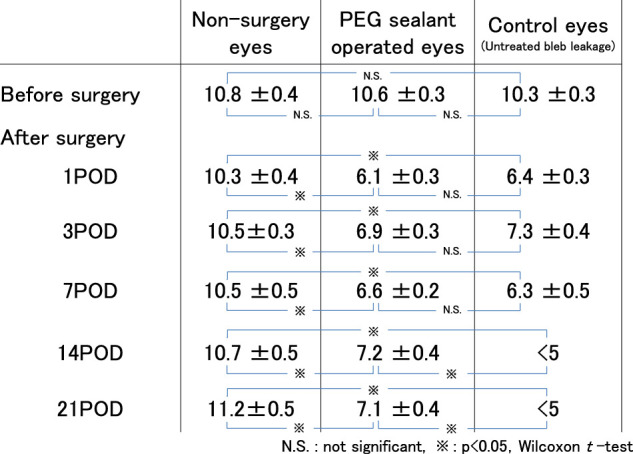

**Figure 3.**
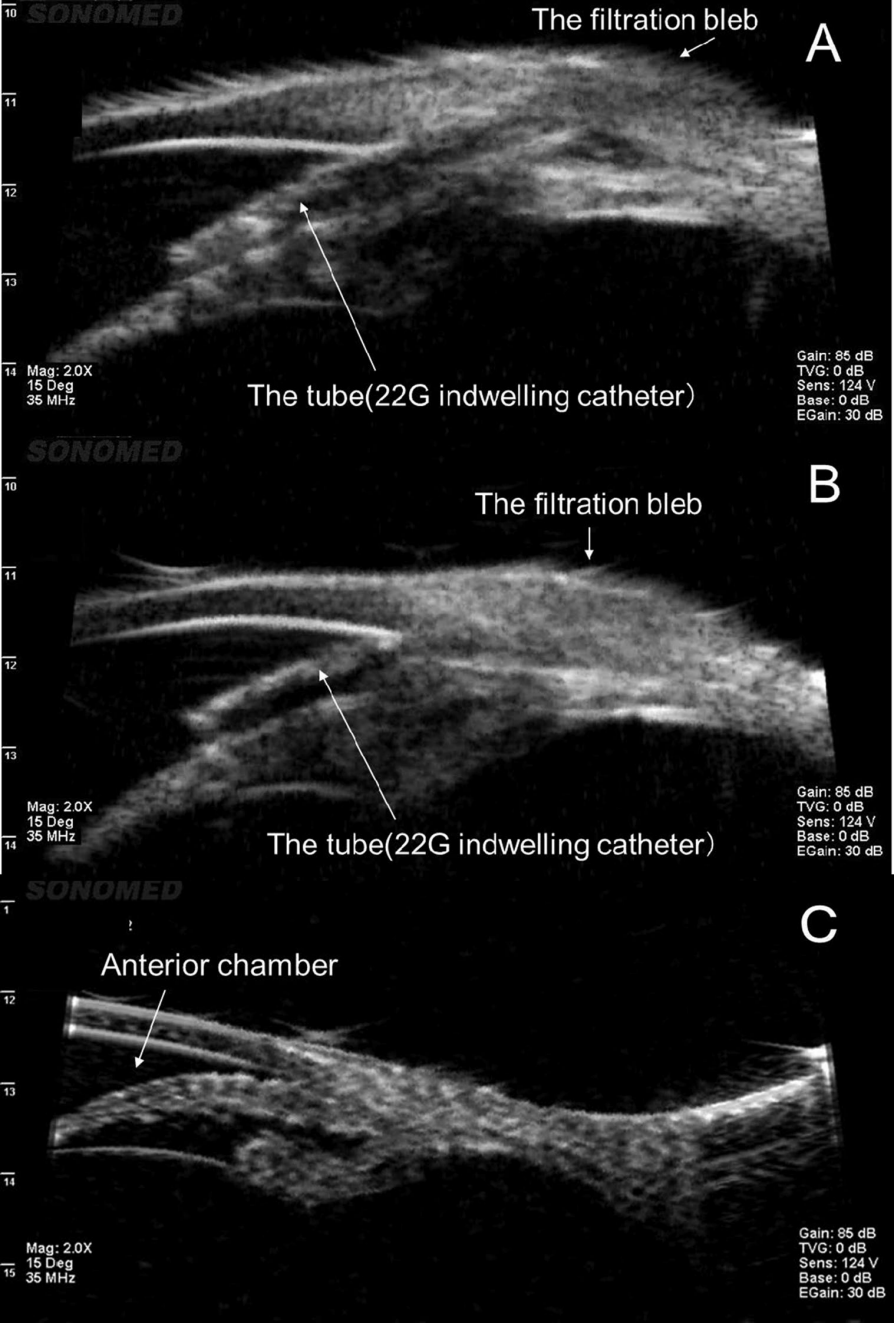
(A) Ultrasound biomicroscopy before bleb perforation. (B) Fourteen days after the PEG sealant is applied to the hole showing that the shape and function of the bleb are maintained after the PEG sealant is applied. (C) Fourteen days after perforation as control of B showing narrow anterior chamber and shriveled filtration bleb.

Ultrasound biomicroscopy showed that after the PEG sealant was applied, the filtration blebs were intact for 14 days after the procedure (Fig. 3).

**Figure 4.**
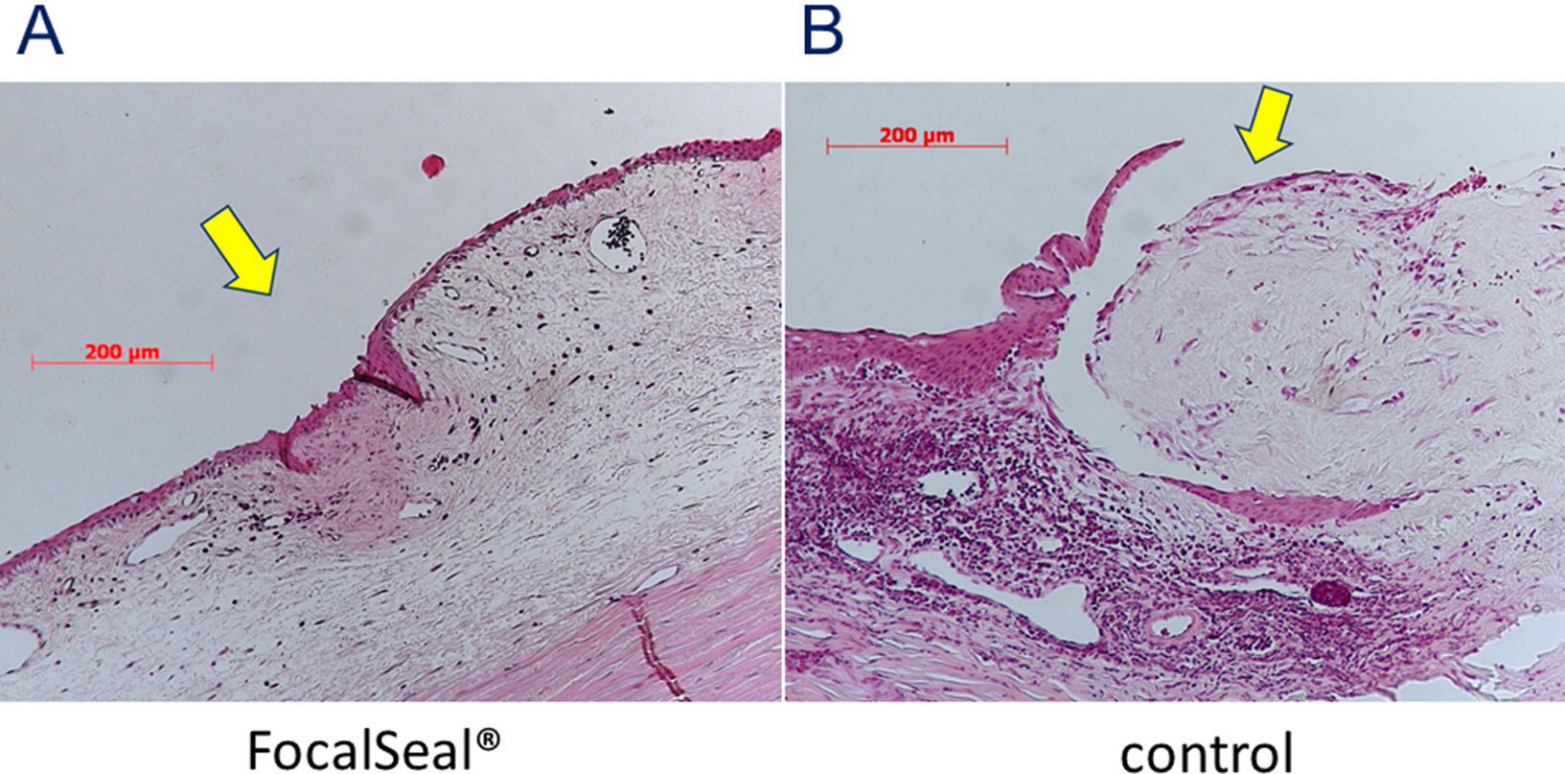
Histologic analysis. (A) HE staining shows that the hole is closed by conjunctival epithelial cells after the PEG sealant application. The arrow indicates the perforation sites. No evidence is seen of excessive inflammatory cells, foreign-body reaction, or toxic effects resulting from application of the PEG sealant. (B) The open hole in the control eye in which the sealant was not applied.

**Figure 5.**
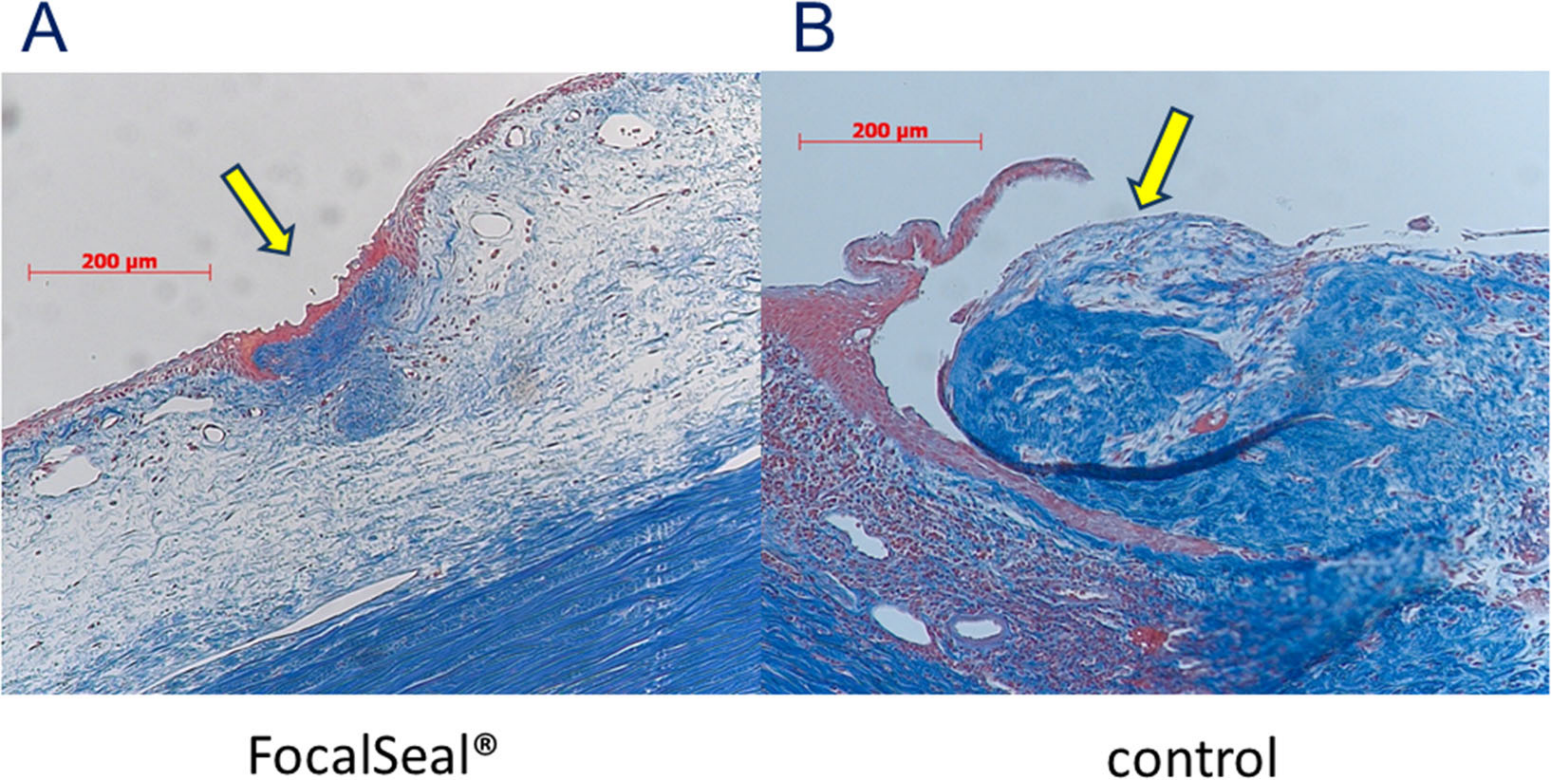
Histologic analysis. Arrows indicate the site of perforation. (A) Masson trichrome staining is very localized. A few fibroblasts and collagen fiber are stained blue in the eye in which the PEG sealant was applied. (B) The fibroblasts and collagen fibers seen extensively in the control group at the perforation site.

**Figure 6.**
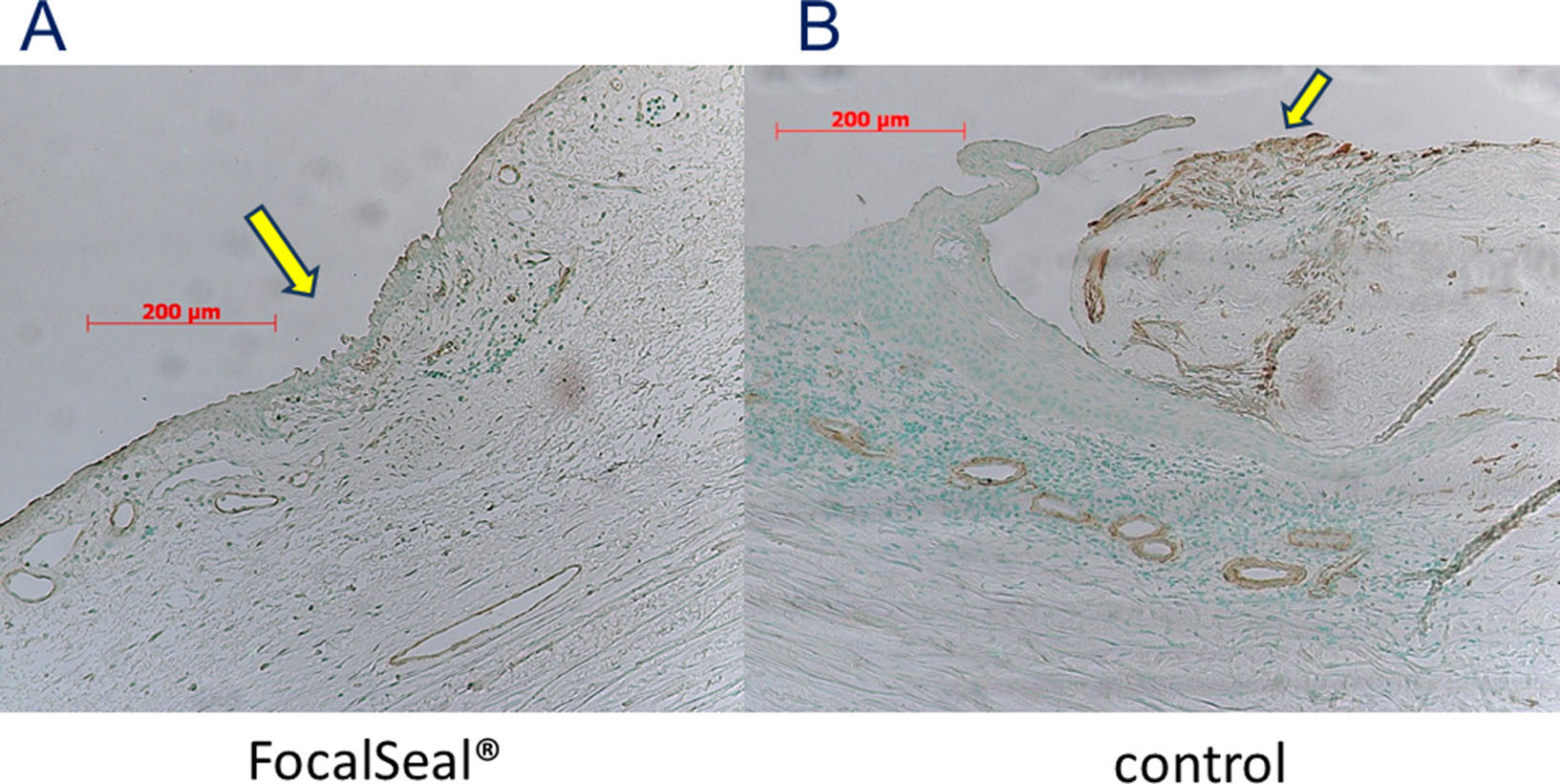
Histologic analysis, α-SMA immunostaining. (A) Few myofibroblasts in the eye in the area where the PEG sealant was applied (arrow). (B) Myofibroblasts developing in the control eye.

HE staining showed that the holes were closed and covered with conjunctival epithelial cells in the eyes in which the sealant was applied (Fig. 4A); the holes were open in the control eyes (Fig. 4B). Masson trichrome staining (Fig. 5) and α-SMA immunostaining (Fig. 6) showed that the bleb holes in which the PEG sealant was used had less fibroblast proliferation and less lymphocytic infiltrates than the holes in which the sealant was not used. Proliferating cell nuclear antigen stain and vimentin stain showed the absence of foreign-body reactions and scarring changes in the blebs in which the sealant was applied. The sealant did not interfere with the wound healing, and no evidence of a significant inflammatory response, fibrosis, or infection with application of the sealant was seen. The healing process was favorable, indicating good biocompatibility.

## Discussion

FocalSeal (Genzyme Corporation) is a water-soluble PEG-based synthetic hydrogel sealant. After polymerization under visible blue-green light, the liquid becomes a clear, solid, flexible, and firmly adherent hydrogel. Because the PEG sealant is not a blood derivative and has been already used in pulmonary^17^^,^^18^ and cardiovascular surgeries,^19^^,^^20^ it is easy to use for ocular clinical applications; general acceptance by the ophthalmic community requires ease of use and safety. For example, fibrin glue^7^^–^^9^ has not been used widely because it is a blood derivative and the associated risks despite desirable results. In our preliminary rabbit studies, we found that 0.2 mL of the subconjunctivally administered PEG sealant was absorbed and disappeared in 4 weeks. In addition, the ophthalmologic and histologic evaluations showed that the PEG sealant had no unfavorable inflammatory effect and did not cause conjunctival or scleral toxicity.

The sealant was applied easily to the bleb holes with a 27-gauge blunt needle and photopolymerized with light. During the sealing procedure, the conjunctival surface around the hole should be dried as much as possible with sterile cotton-tipped applicators or surgical sponges. The PEG sealant appears to be more suitable than cyanoacrylate for closing bleb holes or tears not only because it does not have an odor or require heat but also because it is easy to deliver and apply for rapid gelling flexibility after light application. Furthermore, the PEG sealant does not become rock-hard like cyanoacrylate.

Histologic evaluations showed that the sealant kept a healthy bleb, preventing the development of unexpected synechiae in the bleb. There was less lymphocytic infiltration at the wounds that used the PEG sealant. The results indicated that the mechanism of closure of the bleb hole with the PEG sealant was growth of conjunctival epithelium that covered the hole before the sealant was absorbed without excess inflammation, synechia, and scarring. That is, the sealant effectively repaired the bleb holes without loss of function.

This study had several limitations. First, the long-term effect of bleb retention was not evaluated. Second, the histologic study was not sequential but focused in the important areas. Third, in our model, rabbits were not glaucoma or high IOP models to start with. Further studies are warranted to evaluate the efficacy of the PEG sealant for stopping bleb leaks in a longer term.

## Conclusions

We found that the PEG sealant effectively sealed bleb leaks in this animal model. On histologic analysis, the sealant was biocompatible, and no toxic effects were related to its use. Theoretically, the sealant would be easy to use in human eyes because it received FDA approval for pulmonary surgery, and the sealant does not expose the patient to exogenous blood-borne pathogens compared with fibrin glue. The use of this sealant as an adjunct to conventional sutures would appear a more efficient and helpful method for treating postoperative bleb leaks. Despite the limitations, we believe that the PEG sealant can be an additional useful approach to overcome bleb leaks after filtration surgery.
